# PET imaging of sodium-glucose cotransporters (SGLTs): Unveiling metabolic dynamics in diabetes and oncology

**DOI:** 10.1016/j.molmet.2024.102055

**Published:** 2024-10-23

**Authors:** Konrad Klimek, Xinyu Chen, Takanori Sasaki, Daniel Groener, Rudolf A. Werner, Takahiro Higuchi

**Affiliations:** 1 Goethe University Frankfurt, University Hospital, Department of Nuclear Medicine, Clinic for Radiology and Nuclear Medicine, Frankfurt, Germany; 2 Nuclear Medicine, Faculty of Medicine, University of Augsburg, Augsburg, Germany; 3 Department of Nuclear Medicine and Comprehensive Heart Failure Center, University Hospital Würzburg, Würzburg, Germany; 4 Faculty of Medicine, Dentistry and Pharmaceutical Sciences, Okayama University, Okayama, Japan; 5 DZHK (German Centre for Cardiovascular Research), Partner Site Frankfurt Rhine-Main, Frankfurt, Germany; 6 The Russell H Morgan Department of Radiology and Radiological Sciences, Division of Nuclear Medicine and Molecular Imaging, Johns Hopkins School of Medicine, Baltimore, MD, United States; 7 German Cancer Consortium (DKTK), Partner Site Frankfurt/Mainz and German Cancer Research Center (DKFZ), Heidelberg, Germany

**Keywords:** PET, Sodium-glucose cotransporters, SGLT2 inhibitors, Glucose metabolism, Diabetes mellitus

## Abstract

**Background:**

Sodium-glucose cotransporters (SGLTs) play a crucial role in glucose regulation and are essential therapeutic targets for diabetes management. Recent advancements have leveraged SGLT-targeted PET imaging to examine these transporters' roles in both health and disease.

**Scope of Review:**

This review highlights recent innovations in PET imaging targeting SGLTs, with a particular focus on SGLT-specific radiotracers, such as alpha-methyl-4-deoxy-4-^18^F-fluoro-d-glucopyranoside (Me-4FDG). It emphasizes the advantages of these radiotracers over conventional ^18^F-2-fluoro-2-deoxy-d-glucose (2-FDG) imaging, especially in assessing SGLT activity. Additionally, the review addresses their potential in evaluating the pharmacodynamics of SGLT inhibitors, investigating metabolic changes in diabetes, and staging cancers.

**Major Conclusions:**

SGLT-targeted PET imaging offers promising improvements in diagnostic accuracy and therapeutic planning. The findings underscore the physiological and pathological significance of SGLTs, indicating that this imaging approach could shape future diagnostic and therapeutic strategies in metabolic and oncologic fields.

## Graphical abstract

**Figure:**
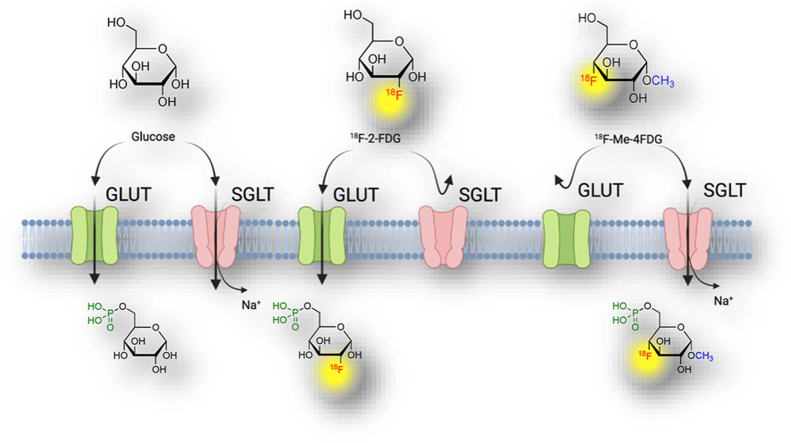

## 1 Introduction

Sodium-glucose cotransporters (SGLTs) belong to a family of membrane proteins pivotal in glucose homeostasis [1,2]. Their primary function is to facilitate the transport of glucose across the cell membrane against its concentration gradient, utilizing the sodium gradient maintained by the Na^+^/K^+^ ATPase pump [3]. The Na^+^/K^+^ ATPase pump actively transports three sodium ions out of the cell and two potassium ions into the cell, creating a high concentration of sodium ions outside the cell compared to inside. This sodium gradient provides the energy needed for glucose transport. SGLTs, specifically SGLT1 and SGLT2, harness this sodium gradient to co-transport sodium and glucose into the cell. In the case of SGLT1, the transport of one glucose molecule is coupled with the transport of two sodium ions, whereas SGLT2 typically couples one glucose molecule with one sodium ion [4]. The transport process begins when sodium ions bind to specific sites on the outward-facing conformation of the SGLT protein, which increases the transporter’s affinity for glucose. Once glucose binds, the transporter undergoes a conformational change, closing the external gate and opening the internal gate, allowing sodium and glucose to be released into the cytoplasm. After releasing the sodium and glucose into the cell, the transporter returns to its original outward-facing conformation to repeat the cycle. This entire process is tightly regulated and reversible, depending on the sodium and glucose gradients across the cell membrane.

In contrast to SGLTs, glucose transporters (GLUTs) function through facilitated diffusion, moving glucose across the cell membrane along its concentration gradient without the need for energy input from ATP [5]. GLUTs are part of the SLC2 family and are ubiquitously expressed in various tissues to regulate glucose uptake according to cellular demand [6]. For example, GLUT4 is insulin-responsive and plays a crucial role in glucose uptake in cardiac muscle and adipose tissue, while GLUT1 ensures basal glucose uptake necessary for cellular metabolism and is widely expressed in tissues with high glucose requirements, such as the brain [7,8].

SGLTs are part of a larger family of transporters SLC5 that include cotransporters for various solutes such as fructose and inositol, and they share structural similarities with other cotransporters and exchangers, such as those in the major facilitator superfamily major facilitator superfamily, which operate using a similar alternating access mechanism [9,10]. In the kidneys, SGLT2 is crucial for reabsorbing glucose from the glomerular filtrate in the proximal tubules, preventing its loss in urine and maintaining blood glucose levels [11,12]. In the intestine, SGLT1 is essential for absorbing dietary glucose, working in concert with other transporters like GLUT2 to ensure efficient nutrient uptake [[12], [13], [14]]. Furthermore, SGLTs are expressed in various tissues, including the heart, brain, and muscle, where they may play additional roles in glucose sensing and cellular protection during metabolic stress. In pathological states such as diabetes mellitus, where SGLT2 inhibitors are therapeutically employed to induce glycosuria and lower plasma glucose levels, the role of SGLTs in glucose homeostasis is especially highlighted [11,12]. In diabetes, the SGLT2 transport capacity is often upregulated, which increases renal glucose reabsorption and exacerbates hyperglycemia. Conversely, SGLT1, while less influential in the kidneys, may compensate for SGLT2 inhibition, underscoring the importance of understanding the interplay between these isoforms. Inhibitors of SGLT2, therefore, offer a unique therapeutic modality by blocking glucose reabsorption, promoting glycosuria, and thus providing an insulin-independent mechanism to lower blood glucose levels [15]. Furthermore, the systemic role of SGLTs extends beyond renal glucose handling and intestinal absorption [16]. These transporters are now recognized for their contribution to the overall glucose economy of the body, influencing the pathophysiology of various conditions, including cardiovascular disease and certain cancers, where altered glucose metabolism is a hallmark [17,18]. Furthermore, SGLT2 inhibitors have been shown to reduce risks of clinical events in patients with heart failure, extending beyond their glucose-lowering effects, though the precise mechanisms remain to be fully elucidated [19,20]. Understanding the role of both SGLT1 and SGLT2 in glucose homeostasis offers profound therapeutic implications, particularly in the context of diabetes management. With the advent of Positron emission tomography (PET) imaging using SGLT-specific radiotracers, it’s now possible to non-invasively explore the functionality of these transporters *in vivo*, providing valuable insights into their biological roles and potential as therapeutic targets.

In this review, we explore the significant advancements in PET imaging using SGLT-specific radiotracers, which provide unprecedented insights into the real-time functioning of SGLTs *in vivo*. These advancements not only allow for a deeper understanding of SGLT activity at the molecular level but also pave the way for innovative diagnostic and therapeutic applications. By enabling precise monitoring of SGLT function in different tissues and organs, PET imaging offers a powerful tool for studying glucose metabolism, enhancing the evaluation of SGLT inhibitors in diabetes treatment, and uncovering their potential role in cancer staging, cardiovascular diseases, and other metabolic disorders.

## 2 Recent advance of PET technology

Recently, PET technology has been glowing with advancements, notably surpassing its nuclear medicine counterpart, Single-photon emission computed tomography (SPECT), in sensitivity and resolution [21,22]. PET’s remarkable ability to detect coincident photon pairs from positron annihilation events eliminates the need for lead collimators, vastly increasing sensitivity and enabling more precise spatial resolution, typically around 4–5 mm. The heightened sensitivity of PET allows for dynamic imaging, offering real-time tracking of radiotracer kinetics for comprehensive functional assessments [23]. Whole-body PET imaging, particularly in oncology, has revolutionized tumor staging and therapy monitoring, significantly impacting patient care [24,25]. PET’s intrinsic technical advantages have made it a preferred modality in many clinical scenarios, supporting a personalized approach to patient care. Efforts to enhance SPECT’s performance continue, but PET’s comprehensive diagnostic capabilities and recent advancements, including the integration of multimodal imaging systems like PET/MRI [26,27] and the introduction of time-of-flight (TOF) technology [28], have solidified its position as a leading tool in nuclear medicine.

PET radionuclides, including Fluorine-18 (^18^F) and Carbon-11 (^11^C), are particularly suitable for small molecule tracers [[29], [30], [31]]. The most common PET tracer, ^18^F-2-fluoro-2-deoxy-d-glucose (2-FDG), has been highly successful for oncological studies due to its ability to highlight areas of increased glucose metabolism, which is a hallmark of many cancers [32]. ^18^F-2-FDG PET is extensively used in diagnosing, staging, and monitoring the treatment response in various malignancies, providing critical information that influences patient management and therapy decisions [33,34]. Recently, the success of oncological staging using ^18^F-2-FDG PET scans has dramatically improved the utilization of PET technology worldwide, underscoring its critical role in modern medical diagnostics.

## 3 PET imaging of SGLT-specific substrate tracers

PET imaging with ^18^F-2-FDG has long been the cornerstone for studying glucose metabolism in various tissues, particularly in oncology [35]. The premise of ^18^F-2-FDG PET imaging relies on the analog nature of ^18^F-2-FDG to glucose, which allows it to be taken up by glucose transporters and phosphorylated by hexokinase [36]. However, unlike glucose, ^18^F-2-FDG is not a substrate for further metabolism and thus accumulates within cells, providing a static snapshot of glucose uptake. The widespread clinical application of ^18^F-2-FDG PET imaging has been crucial in the identification and staging of cancers, given that many tumor cells exhibit an increased glycolytic rate—a phenomenon known as the Warburg effect [37,38]. This increased uptake and trapping of ^18^F-2-FDG in tumor cells render them detectable against the background of normal tissue metabolism, providing valuable diagnostic and prognostic information. Despite its utility for measuring glucose transport, ^18^F-2-FDG is transported into cells predominantly by GLUTs, but it is not a substrate for SGLTs, which play a significant role in renal and intestinal glucose handling and may contribute to the pathophysiology of various diseases. As such, the metabolic activity captured by ^18^F-2-FDG PET does not fully reflect the complexity of glucose dynamics mediated by SGLTs, potentially leading to an underrepresentation of the glucose transport landscape, particularly in non-malignant tissues where SGLT activity is prominent [39,40]. The recognition of these limitations has spurred the development of novel radiotracers that can provide a more comprehensive understanding of glucose metabolism by including the activity of SGLTs. By supplementing the insights gained from traditional ^18^F-2-FDG PET imaging with these new tools, researchers can now investigate glucose metabolism with a broader scope, encompassing both GLUT and SGLT mediated processes, thereby refining the diagnostic accuracy and therapeutic monitoring across a spectrum of diseases.

**Figure 1:**
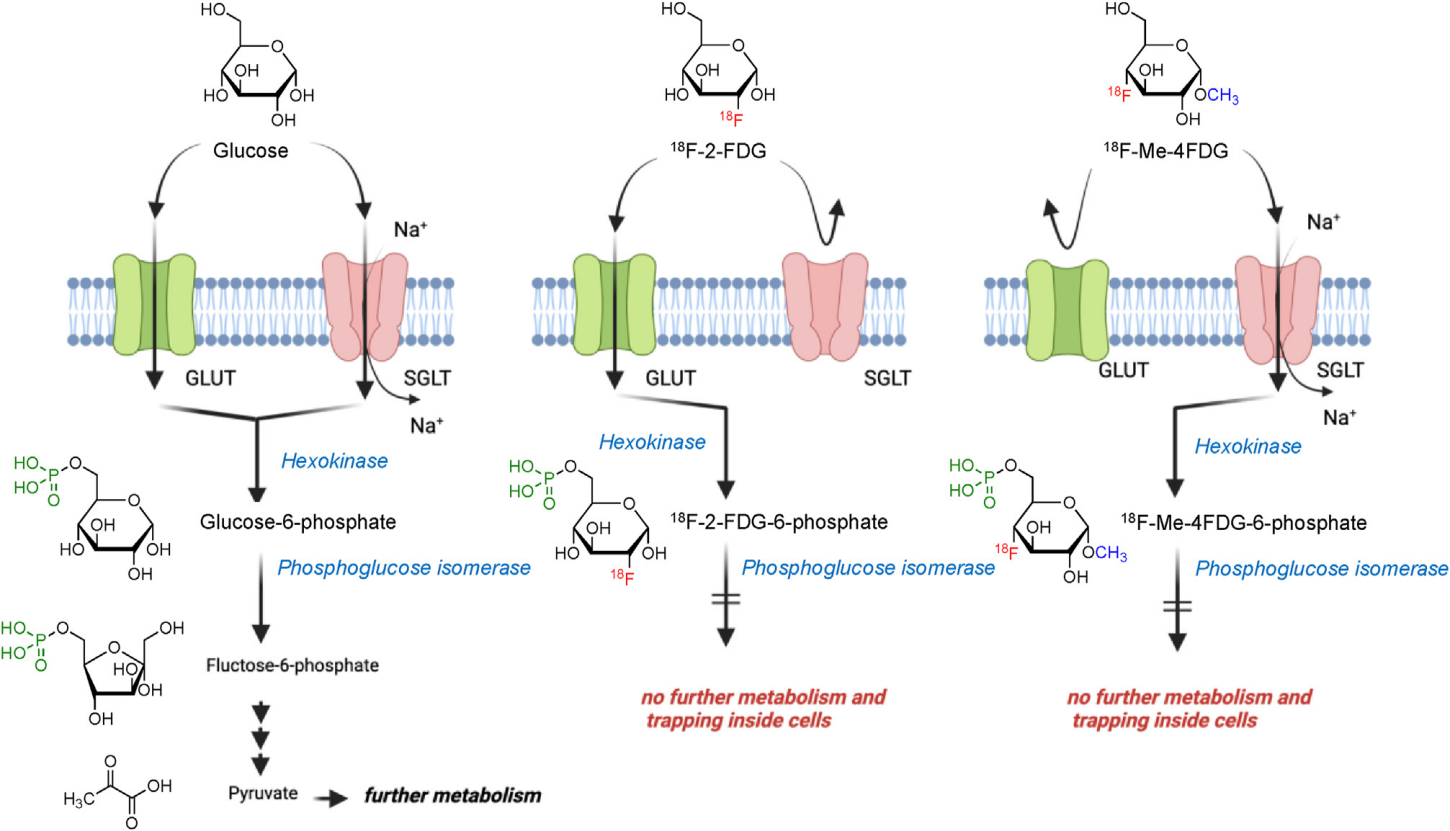
Molecular structures and interactions with glucose transporters (GLUT) and sodium-glucose cotransporters (SGLT), along with the metabolism of glucose and its analogs, ^18^F-2-Fluoro-2-deoxy-d-glucose (^18^F-2-FDG) and alpha-methyl-4-deoxy-4-[18]F-fluoro-d-glucopyranoside (^18^F-Me-4FDG), used as positron emission tomography (PET) tracers. Unlike the physiological substrate glucose, ^18^F-2-FDG is taken up through GLUT but not SGLT. Conversely, ^18^F-Me-4FDG is taken up via SGLT but not GLUT. Once inside the cell, both ^18^F-2-FDG and ^18^F-Me-4FDG are phosphorylated by hexokinase and are resistant to further metabolism by phosphoglucose isomerase, remaining to their intracellular retention. Created with BioRender.com.

**Figure 2:**
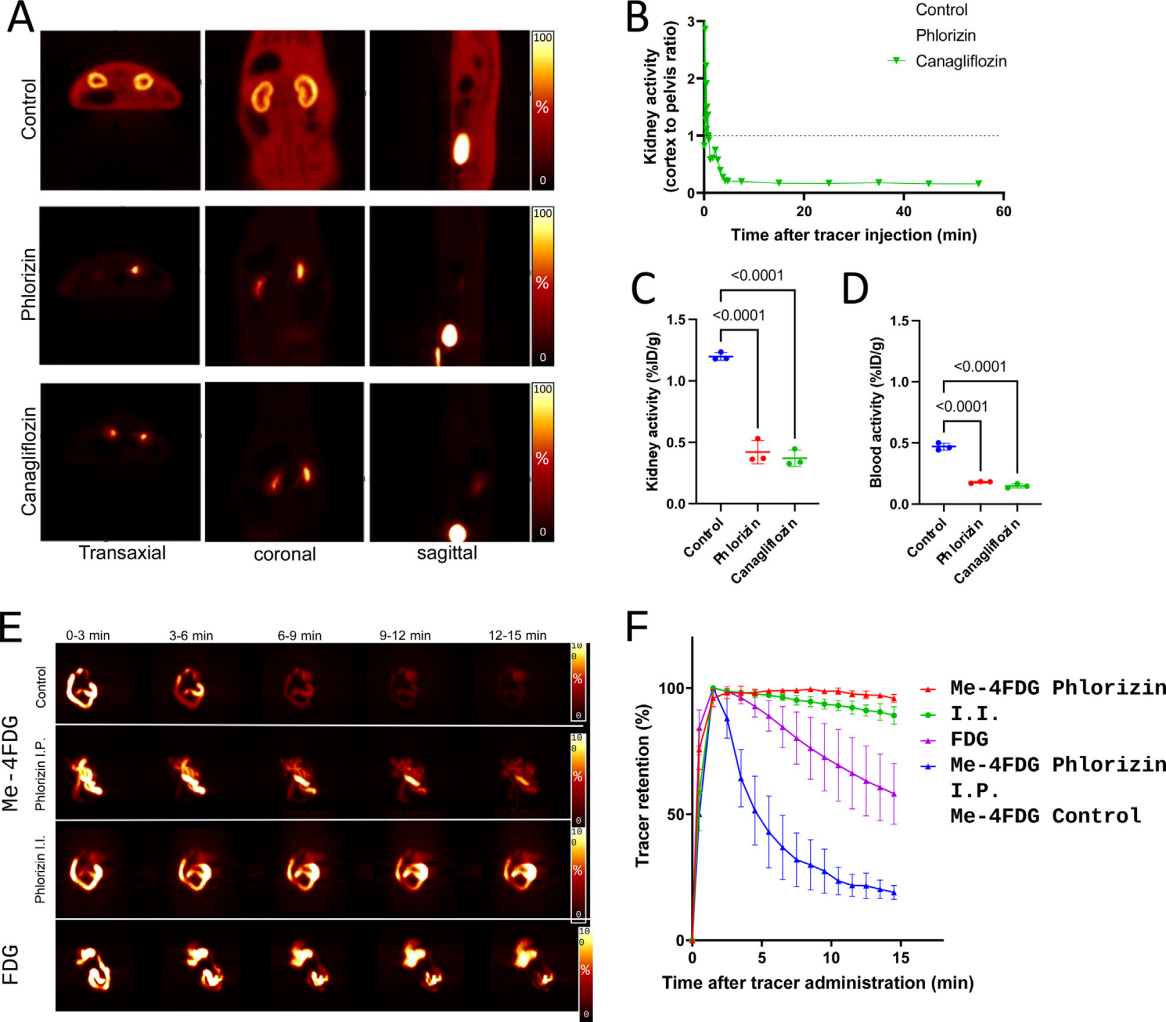
**A.** Renal ^18^F-Me-4FDG PET scans comparing an untreated control rat with pre-treated with phlorizin (nonselective SGLT1/2 inhibitor) and canagliflozin (SGLT2 inhibitor). Control rats (upper rows) showed significant tracer accumulation in the renal cortex. In contrast, rats treated with phlorizin (middle rows) and canagliflozin (lower rows) exhibited markedly reduced activity in the renal cortex and soft tissue, with increased radioactivity noted in the renal pelvis and bladder. **B.** Time-activity curves of renal ^18^F-Me-4FDG PET scans displaying the cortex-to-pelvis count ratios, which show significantly lower renal cortical activity in the pre-treated rats over time. **C.** Comparison of kidney radioactivity at 60 min post-tracer administration across the three groups. **D.** Comparison of blood radioactivity levels at 60 min post-tracer administration. **E.** The series of coronal maximum intensity projection images demonstrate dynamic ^18^F-Me-4FDG PET activity following intra-intestinal administration in rats. The sequence compares untreated controls (upper rows) with those receiving intraperitoneal (I.P., middle rows) and intra-intestinal (I.I., lower rows) phlorizin. Notably, in phlorizin-treated subjects, tracer retention was stable compared to a significant decrease over time in untreated controls. For context, ^18^F-2-FDG images (last column) show very slow clearance. **F.** Time-activity curves of intestinal clearance of tracer illustrate the quantitative differences in radiotracer retention across the groups. Adapted with permission from [42], Copyright 2023, Hindawi.

The advancing frontier of PET imaging has been marked by the groundbreaking advent of SGLT-specific tracers, heralding a new era of *in vivo* metabolic investigation. Pioneering studies utilizing SGLT-specific radiotracers like alpha-methyl-4-deoxy-4-[18]F-fluoro-d-glucopyranoside (^18^F-Me-4FDG) have unveiled novel insights into the multifaceted roles of SGLTs [39,[41], [42], [43], [44]] (Figure 1, Table 1). ^18^F-Me-4FDG’s selectivity for SGLTs allows for a more accurate depiction of glucose dynamics across various tissues, particularly under diabetic conditions where SGLT2 inhibitors serve as crucial therapeutic agents (Figure 2) [42]. Another promising tracer is ^11^C-methyl-D-glucoside (^11^C-MDG), which exhibits affinity for both SGLT1 and SGLT2 [45]. However, the shorter half-life of ^11^C may present challenges for routine clinical application.

**Table 1:** Overview of SGLT transport imaging studies.

Author (year), (ref#)	Tracer	Study design	Summary of findings
Yu et al. (2010), [43]	^18^F-Me-4FDG	Preclinical, rat experiments	Confirmed tracer uptake via SGLTs in specific area of the brain
Yu et al. (2013), [44]	^18^F-Me-4FDG	Preclinical, rat experiments	Regional distribution of functional SGLTs in rat brain
Scafoglio et al. (2015), [39]	^18^F-Me-4FDG	Preclinical, mouse models of cancers	Potential for diagnose and stage pancreatic and prostate cancers
Mitsuoka et al. (2016), [45]	^11^C-MDG	Preclinical, rat experiments	Kidney tracer retention inhibited by SGLT2 inhibitors
Sala-Rabanal et al. (2016), [41]	^18^F-Me-4FDG	Preclinical, mouse models	SGLTs are important in glucose recovery in the kidney
Kepe et al. (2018), [66]	^18^F-Me-4FDG	Clinical, astrocytic tumor (n = 4)	Me-4FDG PET provides high contrast tumor imaging.
Sala-Rabanal et al. (2018), [70]	^18^F-Me-4FDG	Preclinical, mouse models	Oral phlorizin inhibited the fast component of glucose absorption
Scafoglio et al. (2018), [65]	^18^F-Me-4FDG	Preclinical, mouse, lung cancer	Targeting SGLT2 in early lung tumors with Me-4FDG PET imaging
Matsusaka et al. (2022), [42]	^18^F-Me-4FDG	Preclinical, rat experiments	Quantitively monitoring of SGLTs inhibition for absorption and excretion
Geist et al. (2023), [48]	^18^F-Me-4FDG	Clinical, type 2 DM (n = 19)	Me-4FDG PET demonstrate SGLT2-related tracer excretion in patients

**Figure 3:**
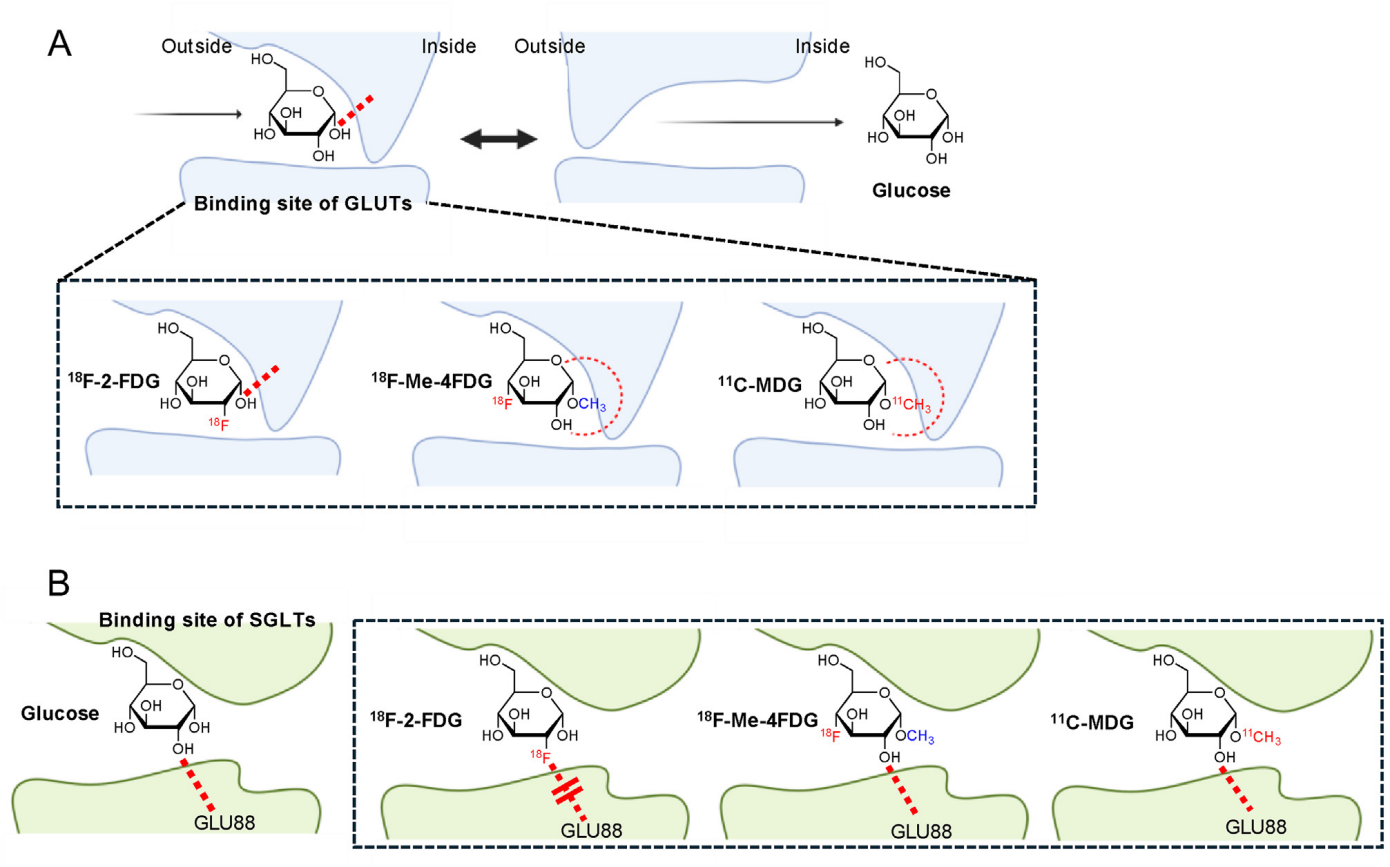
Schematic diagram of the binding sites of GLUTs (A) and SGLTs (B), alongside the molecular structures of the tracers and the proposed hypothesis regarding binding selectivity. In the case of glucose binding to GLUTs, the formation of hydrogen bonds with the C1–OH group is deemed crucial [47], resulting in a high affinity for ^18^F-2-FDG due to the intact structure of C1–OH. In contrast, the affinities decrease for ^18^F-Me-4FDG and ^11^C-MDG when C1–OH is methylated, attributed to spatial limitations. Regarding SGLTs, a hydrogen bond between the amino acid residue GLU88 and the C2–OH group of glucose is identified as crucial [46]. Consequently, the binding affinity weakens for ^18^F-2-FDG due to fluorination at C2, while ^18^F-Me-4FDG and ^11^C-MDG maintain their affinities due to the unchanged C2–OH group, akin to glucose. Created with BioRender.com.

The incorporation of a methyl group in compounds like ^11^C-MDG and ^18^F-Me-4FDG likely enhances their recognition and uptake, potentially due to the larger binding sites of SGLTs [46]. This speculation is based on the notion that the structural flexibility afforded by the methyl group may allow for optimal fitting within the binding site of SGLTs (Figure 3). Conversely, the presence of the methyl group may hinder the interaction of ^11^C-MDG and ^18^F-Me-4FDG with GLUTs [47], which often feature more constrained binding sites. This consideration suggests that GLUTs, primarily responsible for passive glucose transport, may have less flexibility in accommodating bulky substituents like the methyl group, potentially resulting in reduced or negligible uptake of these tracers. The proposed structural specificity conferred by the methyl group may facilitate the precise imaging of tissues exhibiting altered glucose metabolism and provide insights into the functional dynamics and expression patterns of SGLTs within living organisms. Nonetheless, further research is required to conclusively elucidate the underlying mechanisms driving the selectivity of these tracers for SGLTs over GLUTs in molecular imaging applications.

The integration of these innovative tracers with PET imaging is set to revolutionize approach to diagnosing diseases. By mapping SGLT activity, we can potentially develop tailored treatments for diabetes [15,48], cardiovascular conditions [49,50], and certain cancers [51]. This functional imaging can also guide the application of SGLT2 inhibitors, such as empagliflozin and dapagliflozin, which have already demonstrated efficacy in cardiovascular outcomes and renal protection [[52], [53], [54]]. The emergence of SGLT-specific PET tracers represents a significant advancement in metabolic imaging and has the potential to impact patient care profoundly [40,55] (Table 1).

## 4 PET imaging of SGLT in diabetes

SGLT inhibitors have revolutionized the management of type 2 diabetes by reducing hyperglycemia through an insulin-independent mechanism that promotes the excretion of glucose in urine. When monitoring the efficacy of SGLT2 inhibitors in the kidneys, PET imaging with SGLT-specific tracers like ^18^F-Me-4FDG or ^11^C-MDG becomes crucial. As mentioned earlier, unlike 2-FDG, which is a substrate for GLUT transporters and widely used to assess insulin-stimulated glucose uptake, ^18^F-Me-4FDG and ^11^C-MDG are selectively taken up through SGLT transporters, making them suitable for visualizing the activity of SGLT2 inhibitors (Figure 2) [48]. The expression of SGLT2 is particularly high in renal tissues, specifically confined to the apical membrane rather than the basolateral membrane, where only GLUT2 are responsible for further transportation. This delineates the rationale and subcellular mechanism behind radiotracers targeting SGLT for specific uptake, enabling direct assessment of drug action *in vivo*. This specificity enables personalized dosing and treatment optimization, as it provides a direct measure of the impact of SGLT2 inhibitors on renal glucose handling.

**Figure 4:**
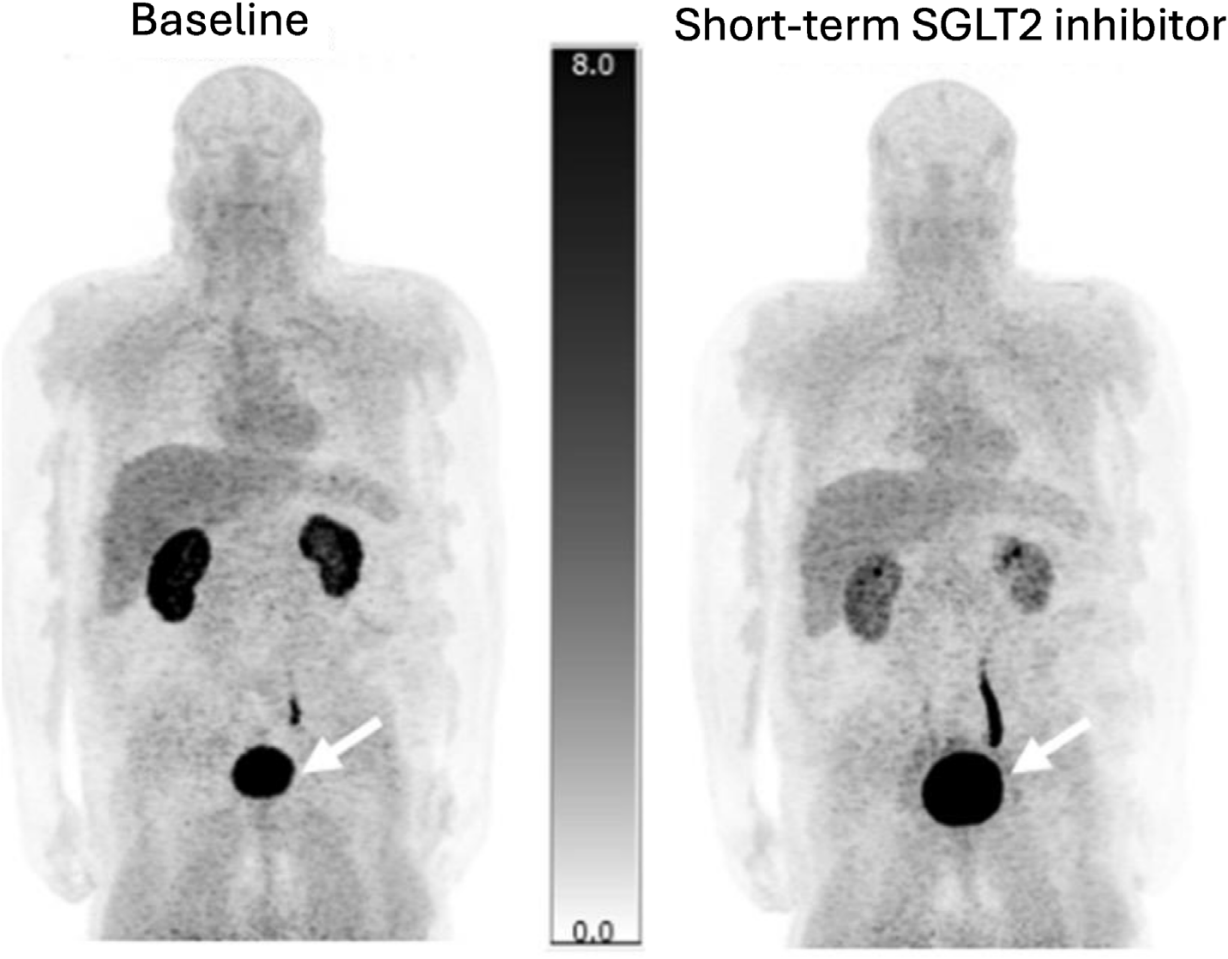
Whole-body PET imaging with ^18^F-Me-4FDG in a type 2 diabetes mellitus patient demonstrates a decrease in renal uptake alongside increased bladder accumulation (indicated by white arrows), comparing baseline (left) and post-2-week treatment with an SGLT2 inhibitor (right). Adapted with permission [48], Copyright 2023, Springer Nature.

Geist et al. investigated whether excretion of ^18^F-Me-4FDG could predict the effectiveness of SGLT2 inhibitor therapy in type 2 diabetes patients [48]. They conducted a longitudinal, prospective study on 19 patients with type 2 diabetes, employing ^18^F-Me-4FDG PET/MRI scans before and after two weeks of SGLT2 inhibitor therapy, while also monitoring blood and urine samples throughout the study. The result revealed that SGLT2 inhibitor therapy significantly increased ^18^F-Me-4FDG excretion (Figure 4) and urinary glucose levels. Additionally, a correlation between baseline urinary glucose and ^18^F-Me-4FDG excretion with the long-term decrease in HbA1c levels was observed. Importantly, ^18^F-Me-4FDG excretion was identified as a predictor for a significant response to SGLT2 inhibitor therapy, while other clinical parameters were not. These findings suggest that the effectiveness of SGLT2 inhibitor therapy in reducing long-term HbA1c levels may be dependent on the body’s endogenous SGLT2 activity. This is the first demonstration using ^18^F-Me-4FDG-PET of the potential of renal SGLT2-related excretion in predicting long-term glycemic control response following SGLT2 inhibitor treatment in type 2 diabetes [48].

## 5 PET imaging of SGLT in oncology

The metabolism of cancer cells is characterized by increased glucose uptake, which is often mediated by upregulated glucose transporter isoforms. While GLUT1 has traditionally been recognized as a key player in cancer glucose metabolism, recent studies have revealed the expression of SGLTs, particularly SGLT1 and SGLT2, in certain cancer types [39,[56], [57], [58], [59], [60], [61], [62], [63]] (Table 2). This broader landscape of glucose transporter expression in cancer cells suggests the potential significance of SGLTs as alternative biomarkers in cancer metabolism. The expression of glucose transporter isoforms, including SGLTs, has been associated with tumor progression and patient survival in various cancer types [64]. While GLUT1 overexpression is commonly observed and correlates with aggressive tumor phenotypes, the role of SGLTs in cancer progression is an emerging area of investigation. Studies exploring the correlation between SGLT expression levels and clinical outcomes could provide valuable insights into the prognostic significance of these transporters in cancer.

**Table 2:** Study overview of SGLT transport expression in various cancers.

Author (Year), (ref #)	SGLT	Cancer Types	Study Design	Summary of findings
Ishikawa et al. (2001), [57]	SGLT-1, -2	Lung Ca.	Analyzed 96 autopsy samples with RT-PCR	Both genes expressed; levels varied between normal and cancer tissues.
Helmke et al. (2004), [58]	SGLT-1	Squamous Cell Ca.	Immunohistochemical analysis of 30 biopsy specimens	Positive staining for SGLT-1 in all samples.
Casnuf et al. (2008), [59]	SGLT-1	Pancreatic Ca.	Examined 83 pancreatic adenocarcinoma specimens	No SGLT-1 staining in normal pancreas; varied scores in cancer.
Lai et al. (2012), [60]	SGLT-1	Ovarian Ca.	Stained 178 ovarian tumor specimens	High expression in 39.7% of invasive carcinomas.
Scafooglio et al. (2015), [39]	SGLT-1, -2	Pancreatic Ca., Prostate Ca.	Mapped distribution in tumors	Both transporters expressed in adenocarcinomas, minimal staining in normal tissue.
Zhou et al. (2020), [61]	SGLT-2	Breast Ca.	Immunohistochemistry and immunoblot of 25 cases	SGLT-2 expressed in cell lines and tissue samples.
Du et al. (2022), [62]	SGLT-1, -2	Pancreatic Ca.	Study with 88 patients, specimens obtained through surgical resection	SGLT-1 overexpression predicts better prognosis than SGLT-2.
Tsunokake et al. (2023), [63]	SGLT-1, -2	Breast Ca.	Analyzed 162 cases of invasive ductal carcinoma	No significant prognostic difference between SGLT-1 and SGLT-2.

Scafoglio et al. identified SGLT2 as a potential marker for early lung tumorigenesis, specifically in premalignant lesions and early-stage lung adenocarcinoma [65]. Using immunohistochemistry and PET imaging with Me-4FDG in mouse models, researchers detected high SGLT2 expression and activity in lung premalignancy and low-grade adenocarcinomas. Moreover, the use of FDA-approved SGLT2 inhibitors significantly inhibited tumor growth and prolonged survival in lung cancer models, suggesting that SGLT2 could serve not only as a diagnostic marker but also as a therapeutic target for early-stage lung cancer.

Taira et al. addressed the issue of false-negative results in mediastinal nodal staging of non-small cell lung cancer during 2-FDG PET/CT scans [64]. The researchers compared the expression of GLUT1, SGLT1, and SGLT2 in false-negative and true-positive lymph nodes. They found that lower GLUT1 expression and higher SGLT2 expression were significantly associated with false-negative metastatic nodes. This finding indicates that the expression levels of these transporters could be contributing to inaccuracies in current ^18^F-2-FDG PET/CT diagnostic methods and could serve as additional markers to improve the staging accuracy in non-small cell lung cancer [64]. These studies underscore the importance of glucose transporters in lung cancer and suggest that incorporating the analysis of SGLT2 expression, along with other transporters like GLUT1, could refine diagnostic imaging techniques and offer new avenues for treatment. The findings prompt further investigation into glucose transporters as biomarkers and therapeutic targets in lung cancer management.

**Figure 5:**
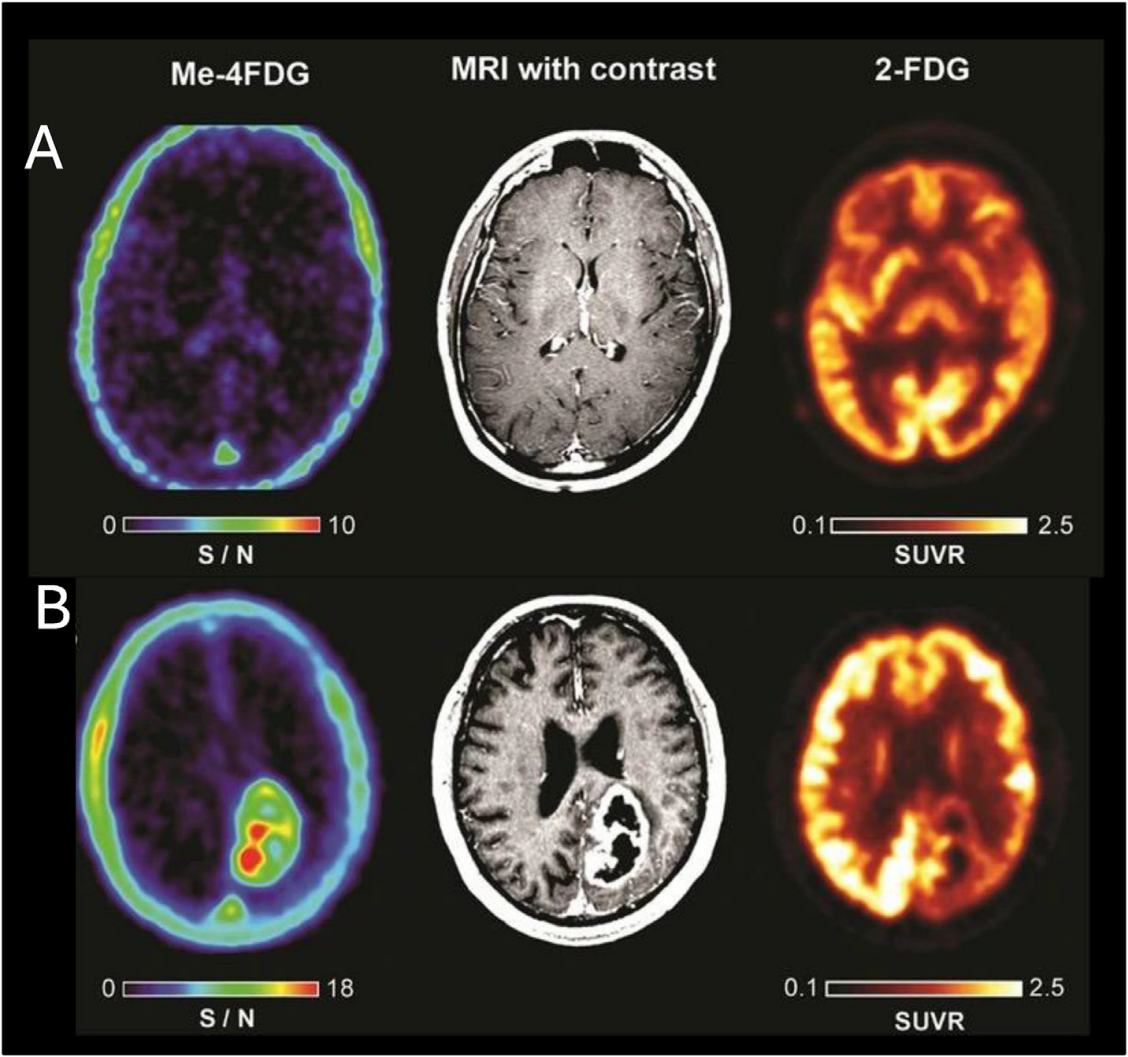
**A.**^18^F-Me-4FDG PET (left), MRI(middle), and ^18^F-2-FDG PET (right) scans on control subject. **B.** images of a patient with posterior para median left parietal lobe glioblastoma. The images illustrate that ^18^F-Me-4FDG PET scans are more effective than ^18^F-2-FDG PET scans in delineating tumor lesions in the patient. Adapted with permission [66], Copyright 2018, Springer Nature.

Kepe et al. evaluated the use of PET imaging to detect brain high-grade astrocytomas by targeting the SGLT2 with ^18^F-Me-4FDG in four patients diagnosed with WHO Grade III or IV astrocytomas [66]. These scans were compared to ^18^F-2-FDG PET scans and MRI. ^18^F-Me-4FDG had a significantly higher uptake in astrocytomas compared to normal brain tissue, providing a high signal-to-noise ratio that may enhance the detection and visualization of these tumors [66] (Figure 5). The distribution of ^18^F-Me-4FDG within the tumors was similar to that of ^18^F-2-FDG, but the distinct contrast provided by the absence of ^18^F-Me-4FDG in the background brain tissue suggested a superior imaging sensitivity. Furthermore, immunocytochemistry indicated that SGLT2 proteins were present in the tumor cells and the endothelial cells of the tumor’s microvasculature. This discovery also points to a potential new therapeutic approach, as SGLT2 inhibitors, already FDA-approved for treating type 2 diabetes, could be repurposed for treating high-grade astrocytomas.

## 6 Future directions

Firstly, there is a significant potential in utilizing ^18^F-Me-4FDG SGLTs imaging to deepen our understanding of glucose metabolism in diabetes and oncology. ^18^F-Me-4FDG PET has already shown effectiveness in these areas, but further exploration in larger clinical trials is needed to elucidate its full clinical utility. Investigating its use in personalized medicine through PET approaches could unlock valuable insights into disease mechanisms and treatment responses.

**Figure 6:**
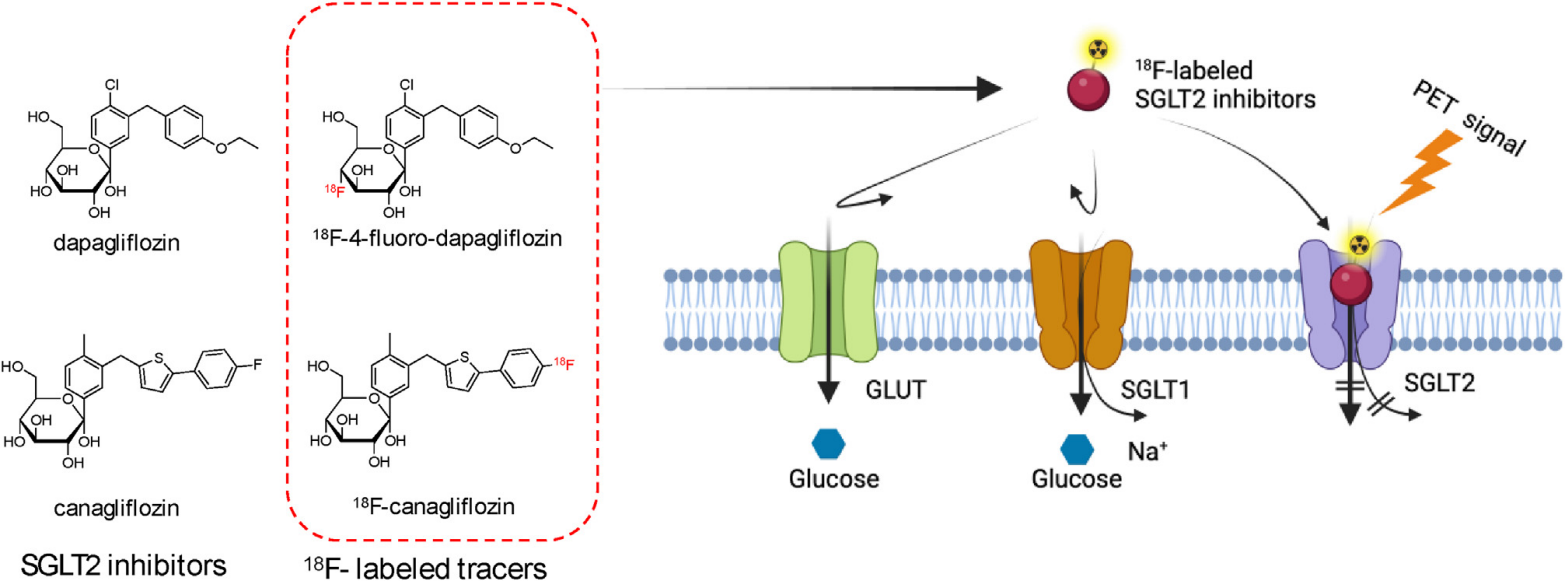
Molecular structures and interactions with GLUT and SGLT of SGLT2 inhibitors: ^18^F-4-fluoro-dapagliflozin [67] and ^18^F-canagliflozin [68] as SGLT2 isoform selective PET tracers. Created with BioRender.com.

Secondly, there is a demand to explore the development of new tracers specifically targeting the SGLT2 isoform. Recent studies in isoform-specific SGLT imaging have yielded significant developments, notably the introduction of targeted PET tracers, such as ^18^F-4-fluoro-dapagliflozin [67] and ^18^F-canagliflozin [68] (Figure 6). These tracers have a structural resemblance to glucose yet are chemically modified to bind selectively to SGLT2 transporters. These chemical alterations are meticulously designed to interact with unique amino acids in the SGLT2 transporter’s binding site—amino acids that are distinct from those in GLUTs or SGLT1. This specificity enhances their effectiveness as imaging agents. Initially developed as SGLT2 inhibitor drugs for diabetes management, these compounds have been successfully repurposed with ^18^F labeling, enabling their use in PET imaging to potentially improve the monitoring and management of diabetes.

Ghezzi et al. utilized PET with ^18^F-4-fluoro-dapagliflozin to investigate the distribution of functional SGLT2 proteins in rodents [67]. Their findings revealed prominent binding of ^18^F-4-fluoro-dapagliflozin in the kidney cortexes of rats and wild-type mice, demonstrating specificity for SGLT2. Microscopic analysis further confirmed binding to the apical surface of early proximal tubules, suggesting its potential as an SGLT2-specific PET tracer. Furthermore, van der Hoek et al. focused on synthesizing ^18^F-canagliflozin, a PET tracer targeting SGLT2, using a Cu-mediated ^18^F-fluorination approach [68]. They carried out a feasibility study using the ^18^F-canagliflozin PET imaging to assess the association between clinical canagliflozin doses and SGLT2 occupancy in patients with type 2 diabetes, providing valuable insights into drug distribution *in vivo* [69]. While Me-4FDG and other SGLT2-targeting PET tracers have shown promise, the development of more specific and sensitive SGLT2 tracers could enable more accurate diagnoses and personalized treatment strategies for conditions like diabetes and cancer.

## 7 Conclusion

Looking ahead, SGLT PET imaging holds considerable promise for advancing personalized medicine in the fields of diabetes and oncology. By providing non-invasive assessments of glucose metabolism at the molecular level, SGLT PET imaging can aid in disease diagnosis, treatment selection, and response monitoring. In diabetes management, SGLT PET imaging may facilitate the optimization of glucose-lowering therapies and the identification of novel therapeutic targets. Similarly, in cancer treatment, SGLT PET imaging has the potential to guide the development of targeted therapies and improve patient outcomes through early detection and treatment monitoring. Continued research efforts aimed at refining imaging techniques, expanding tracer availability, and addressing ethical and regulatory considerations are essential for realizing the full clinical impact of SGLT PET imaging in diabetes and cancer treatment.

## Funding

This project is partially supported by the 10.13039/501100012330Okayama University “RECTOR” Program, 10.13039/501100001691KAKENHI grant (22H03027) from the 10.13039/501100001691Japan Society for the Promotion of Science (T.H.) and the 10.13039/501100001659German Research Foundation (453989101, R.A.W., T.H.; 507803309, R.A.W.).

## Research involving human participants or animals

This article does not describe any studies with human participants or animals performed by any of the authors.

## Others

ChatGPT has been employed for editing tasks.

## CRediT authorship contribution statement

**Konrad Klimek:** Writing – review & editing, Writing – original draft, Visualization. **Xinyu Chen:** Writing – review & editing, Writing – original draft, Conceptualization. **Takanori Sasaki:** Writing – review & editing, Writing – original draft, Data curation, Conceptualization. **Daniel Groener:** Writing – review & editing, Writing – original draft, Conceptualization. **Rudolf A. Werner:** Writing – review & editing, Writing – original draft, Conceptualization. **Takahiro Higuchi:** Writing – review & editing, Writing – original draft, Visualization, Validation, Supervision.

## Declaration of competing interest

The authors declare that they have no known competing financial interests or personal relationships that could have appeared to influence the work reported in this paper.

## Data Availability

No data was used for the research described in the article.
